# Synthetic Strategies for 5- and 6-Membered Ring Azaheterocycles Facilitated by Iminyl Radicals

**DOI:** 10.3390/molecules21050660

**Published:** 2016-05-18

**Authors:** John C. Walton

**Affiliations:** University of St. Andrews, EaStCHEM School of Chemistry, St. Andrews, Fife KY16 9ST, UK; jcw@st-andrews.ac.uk; Tel.: +44-1334-463-864; Fax: +44-1334-463-808

**Keywords:** organic synthesis, radicals, heterocycles, photoredox catalysis, natural products

## Abstract

The totality of chemical space is so immense that only a small fraction can ever be explored. Computational searching has indicated that bioactivity is associated with a comparatively small number of ring-containing structures. Pyrrole, indole, pyridine, quinoline, quinazoline and related 6-membered ring-containing aza-arenes figure prominently. This review focuses on the search for fast, efficient and environmentally friendly preparative methods for these rings with specific emphasis on iminyl radical-mediated procedures. Oxime derivatives, particularly oxime esters and oxime ethers, are attractive precursors for these radicals. Their use is described in conventional thermolytic, microwave-assisted and UV-vis based preparative procedures. Photoredox-catalyzed protocols involving designer oxime ethers are also covered. Choice can be made amongst these synthetic strategies for a wide variety of 5- and 6-membered ring heterocycles including phenanthridine and related aza-arenes. Applications to selected natural products and bioactive molecules, including trispheridine, vasconine, luotonin A and rutaecarpine, are included.

## 1. Introduction

Several recent articles have drawn attention to the virtually boundless extent of chemical space; the domain that contains the totality of all possible compounds [1,2]. The total number of molecules that could be made from only 30 atoms is in the range 10^20^ to 10^24^ [3], “drug-like” chemical space comprises over 10^60^ molecules [4,5] and, of course, even these huge numbers are insignificant in comparison with the protein or nucleic acid spaces. The number of polypeptide chains of modest (250 unit) length, drawn from the 20 natural amino acids, exceeds the ‘trans-astronomical’ number of 10^325^ [6]. The CAS registry currently contains about 10^8^ chemical substances. Its present rate of growth is about 5 × 10^6^ substances per year, so that at this rate more than 10^54^ years would be needed just to explore “drug-like” chemical space! It is abundantly evident that only a minute fraction of chemical space can ever be preparatively accessed. To address this problem, computational algorithms are being devised capable of virtual screening and/or for locating, within the total space, “islands” or “trees” of substances with potentially desirable properties such as bioactivity [1,2,7]. Ertl and co-workers developed self-organising neural networks which showed that a comparatively small number of ring structural units is associated with bioactivity. They listed 30 heterocyclic moieties as of crucial importance and 22 of these contained one or more N-atoms [7]. Pyrrole, indole and related structures figured prominently, as did pyridine, quinoline, quinazoline and analogous 6-membered ring-containing aza-arenes. The immense size of chemical space presents exciting opportunities of discovering hitherto unknown and extraordinary substances with properties beneficial to human society. Its size also represents a huge challenge for preparative chemists such that it is imperative to open up every possible avenue that might facilitate the task. The exploration and exploitation of identified “islands” depends critically, of course, on the availability of practical preparative methods. Thus, the development of synthetic strategies for the ring systems associated with bioactivity that are fast, efficient and of low environmental impact, deserves special attention.

The advantages of free-radical based preparative methods include the usually neutral conditions, the tolerance for many unprotected functional groups and the availability of much kinetic, thermodynamic and mechanistic data to guide the design of experimental methodology. During the last two decades a great deal of research has been directed towards making radical-mediated synthetic methods safer, more efficient and more convenient [8,9,10,11,12,13]. New tactics have been devised for avoiding hazardous initiator peroxides or azo-compounds and for dispensing with toxic tin, mercury, copper and other metal reagents. For example, ‘pro-aromatic’ reagents, based on the cyclohexadiene structure, release many radical types without the need for metals [14]. Murphy and co-workers’ development of organic super electron donors unlocked completely new ways of generating radicals and radical-ions and harnessing them synthetically [15,16,17]. The unique properties of organoboron compounds have led to the design of several different reagent types for radical release including *B*-alkylcatecholboranes [18,19] and N-heterocyclic carbene boranes [20,21,22,23]. The discovery of homogeneous photoredox catalysts (PCs) has had huge impact on radical-mediated preparations. The most popular are complexes of Ru or Ir [24,25,26,27] that re-introduce metals, albeit in small quantities. However, organic dyes and other donor molecules are also coming into use as PCs [28,29]. Heterogeneous photoredox catalysts, particularly titanium dioxide (titania, TiO_2_), possess the added convenience of easy removal after use by filtration or centrifugation. Their exploitation for radical mediated preparations is also developing rapidly [30,31,32].

The N–O bonds in oximes and in oxime derivatives are comparatively weak and break homolytically with production of a pair of N- and O-centered radicals. Aldehydes and ketones are available as starting materials in huge variety from natural and commercial sources. Oximes can be prepared essentially quantitatively from them simply by treatment with hydroxylamine hydrochloride. The consequence is that oxime esters of many types containing the >C=N–OC(O)– structural unit (carbonyl oximes) and oxime ethers containing the >C=N–O– unit are very readily accessible. Most members of both classes are stable to moderate heat and hydrolysis, are non-toxic and non-hazardous, are easily handled and have long shelf lives. Suitably functionalised, they have proved amazingly adaptable for radical generation by an unprecedentedly wide range of methods [33]. These include conventional thermolyses, microwave irradiations, UV photolyses, sensitised UV photolyses and with several types of photoredox catalysis. Oxime derivatives are therefore particularly flexible, convenient and benign and stand as very attractive alternatives to other more hazardous radical precursors. This article reviews the use of both carbonyl oximes and oxime ethers in radical mediated organic syntheses.

**Scheme 1:**
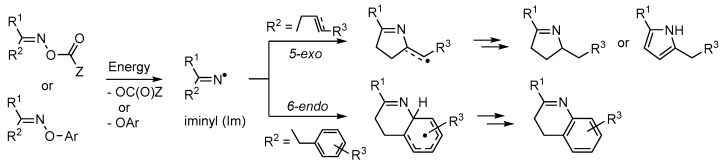
Generation of iminyl radicals from oxime derivatives and subsequent ring closures.

When fittingly stimulated, both compound types initially yield N-centred iminyl radicals >C=N^•^ (Im, Scheme 1). Those suitably accoutred with acceptor groups can, when appropriately manipulated, yield azaheterocycles. An O-centred radical [^•^OC(O)Z] is released from a carbonyl oxime together with the iminyl radical and can be chosen to end up as volatile or otherwise easily separable by-products. For the oxime ether precursors, best results are usually achieved with *O*-aryl substituents. In this case the by-product is usually a phenol (ArOH) which can readily be removed because of its mild acidity. Iminyl radicals with butene or butyne type side chains selectively undergo *5-exo* cyclisation to produce 5-member ring containing dihydropyrrole type products. By way of contrast, iminyl radicals with aromatic or heteroaromatic acceptor substituents preferentially yield 6-membered ring pyridine, quinoline *etc.* products. In some instances this results from an initial *5-exo spiro* cyclization followed by ring expansion via an aziridinyl type intermediate (see for example Section 4.1). Preparations of many different azaheterocycle types may therefore be achieved by careful choice of the acceptor substituent(s), and by tuning the reaction conditions and methodology.

This review also focuses on the iminyl radical based synthetic methodology developed for the sets of aza-arenes identified above as of importance in the bioactivity islands of chemical space.

## 2. Syntheses of Dihydropyrroles and Pyrroles

Organotin promoted radical methodology is justly famed because it works seamlessly in so many situations and has proved so dependable. Many ingenious syntheses of azaheterocycles have employed organotin hydrides or ditins for the generation of iminyl or aminyl radicals. Zard, for example, described tin hydride-mediated syntheses of dihydropyrroles, indolizidines and other aza-heterocycles from sulfenimines (PhS–N=C<), thionocarbazones and other derivatives [34,35]. Nanni and co-workers generated iminyl radicals by ring closures of C-centred radicals onto organic nitriles and hence prepared many heterocyclic systems. They also employed tin-free thermolytic and other processes [36,37,38]. Much of this earlier research has been reviewed by Bowman and Aldabbagh [39,40,41] and/or by Fallis and Brinza [42]. Recently, Zhang and Studer have published an outstanding review of aza-arene syntheses flowing from radical additions to organic isonitriles [43]. This methodology exploits the formation and ring closures of amidoyl radicals (Ar–N=C^•^–R).

### 2.1. Pyrrole and Dihydropyrrole Preparations from Carbonyl Oximes

**Scheme 2:**
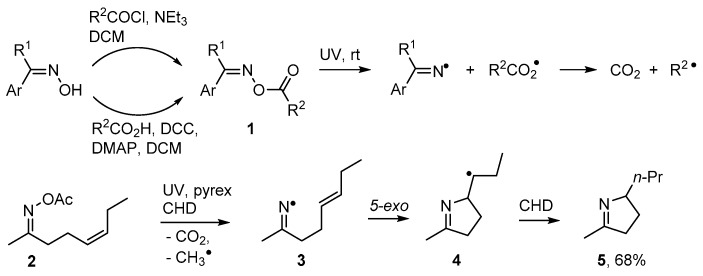
Photo-dissociation of oxime esters and preparation of dihydropyrroles [48,51].

Numerous alkaloids contain pyrrole, dihydropyrrole or related rings and many biological roles are associated with these structures [44,45,46]. Oxime esters **1** can easily be prepared from oximes reacting with either carboxylic acids or acyl halides [47,48]. On photolysis they release an iminyl radical together with an acyloxyl radical and the latter rapidly extrudes CO_2_ with production of a C-centred radical [48,49] (Scheme 2). Rodrigues, Sampedro and co-workers used acyl oximes such as **2** as efficient sources of iminyl radicals [50,51,52]. With this precursor type, the radical co-produced with the iminyl during UV photolyses was acyloxyl [CH_3_CO_2_^•^] that simply furnished volatile CH_4_ and CO_2_ as by-products. They reported that the iminyls could be conscripted into syntheses of many types of heterocycles including dihydropyrroles. UV photolysis of **2** through Pyrex released iminyl radical **3** with an alkenyl side chain. These selectively cyclised in the *5-exo* mode with production of pyrolidinylmethyl radical **4** that subsequently abstracted an H-atom from co-reactant cyclohexa-1,4-diene (CHD) to yield 3,4-dihydropyrrole derivative **5** (Scheme 2).

**Scheme 3:**
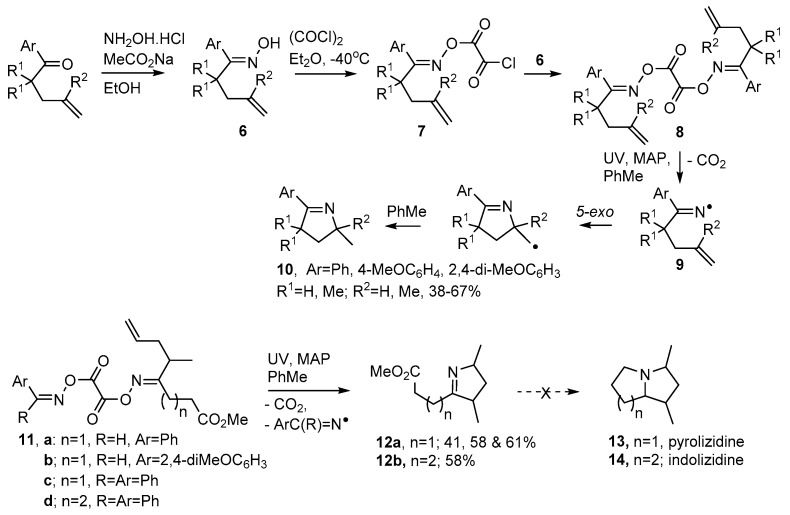
Preparation of dioxime oxalates and their use in dihydropyrrole syntheses [54].

Dioxime oxalates **8** are another type of oxime ester that proved convenient for clean generation of iminyl radicals. Symmetrical types were made by treatment of an oxime **6** with oxalyl chloride to yield oxime oxalyl chlorides **7** as intermediates (Scheme 3). Although these could be isolated, they hydrolysed and degraded quickly, so immediate treatment with another equivalent of either the same oxime **6**, or a different oxime, yielded the symmetrical dioxime oxalates **8** or unsymmetrical types such as **11a**–**d** respectively [53,54]. Dioxime oxalates **8** were “clean” and atom-efficient because, apart from CO_2_, they delivered only iminyl radicals **9** on UV photolysis. Photo-dissociations were aided by inclusion of 4-methoxyacetophenone (MAP) as photosensitizer and by the presence of an aryl substituent adjacent to the C=N bond.

3,4-Dihydropyrrole derivatives **10** were obtained in moderate yields with toluene acting as both solvent and H-atom donor (Scheme 3). Unsymmetrical dioxime oxalates such as **11** incorporated an aryl-oxime unit, to promote photo-dissociation, as well as an alkenic acceptor unit. These precursors enabled non-aromatic dihydropyrroles such as **12a**,**b** to be accessed. Attempts to convert the latter to pyrolizidines **13** and indolizidines **14** were unsuccessful.

### 2.2. Pyrrole and Dihydropyrrole Preparations from Oxime Ethers

Thermal preparative methods are often superior because of their simplicity and non-hazardous nature. *O*-Phenyl oxime ethers **15** can easily be made by treatment of carbonyl compounds with the commercially available *O*-phenylhydroxylamine hydrochloride. Conventional thermolyses of appropriate derivatives were shown to provide dialkyl- or diaryl-iminyl radicals [55]. Subsequently it was established that microwave heating (μwave) was a particularly efficient means of releasing iminyl radicals and mediating dihydropyrrole preparations [56,57].

**Scheme 4:**
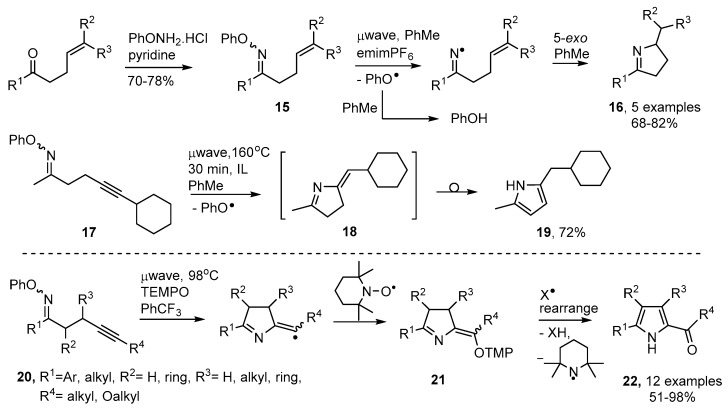
Preparation of oxime ethers and microwave-promoted syntheses of dihydropyrroles and pyrroles [56,57,58].

The optimum procedure utilized toluene as both solvent and H-donor together with an equivalent of the ionic liquid (IL) 1-ethyl-3-methyl-*1H*-imidazol-3-ium hexafluorophosphate (emimPF_6_) to promote microwave absorbance. This method enabled ketones with but-3-enyl type side chains to be converted to dihydropyrroles **16** in good yields in two steps (Scheme 4). The phenoxyl radicals released from **15** also abstracted H-atoms from the solvent to afford phenol as an easily separable by-product. When oxime ether **17** with an alkyne side chain was microwave irradiated under similar conditions, pyrrole **19** was isolated in good yield. Evidently the first-formed methylene-dihydropyrrole **18** rearranged under the reaction conditions.

Castle and co-workers prepared a set of alkyne-substituted oxime ethers **20** and carried out microwave irradiations of mixtures with tetramethylpiperidine-*N*-oxide (TEMPO) in benzotrifluoride solvent [58]. The ring closed radicals were trapped by the TEMPO with production of intermediates **21** (Scheme 4). These also rearranged, with loss of a piperidinyl radical, so providing 2-acylpyrroles **22** in good to excellent yields.

### 2.3. Photoredox Catalyzed Preparations of Pyrroles and Dihydropyrroles from Oxime Derivatives

**Scheme 5:**
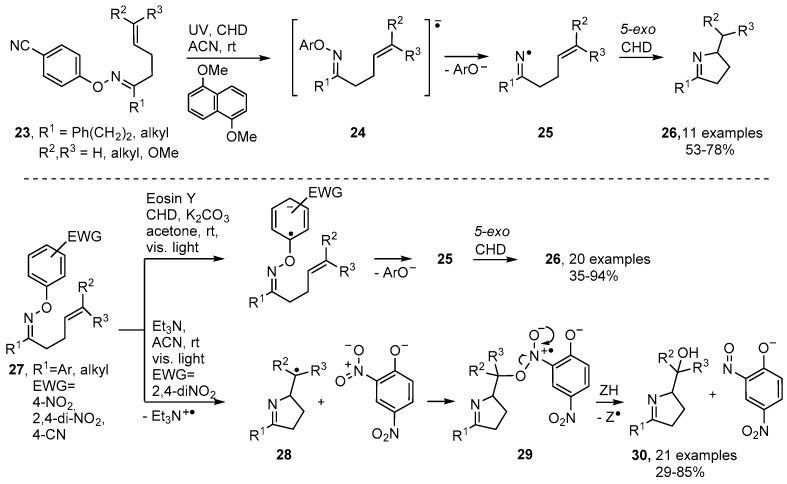
Photoredox catalytic routes from oxime ethers to dihydropyrroles [28,60].

Photoredox catalytic methodology [25,27,59] has also been developed for production of dihydropyrroles from *O*-aryl oxime ethers substituted with electron withdrawing groups (EWG) in their *O*-aryl units. Narasaka and co-workers prepared oxime ethers **23** containing 4-CN (or 2,4-di-NO_2_ or 4-CF_3_) aryl substituents. On inclusion of a catalytic amount of 1,5-dimethoxynaphthalene (DMN) and irradiation with UV light in 1,4-cyclohexadiene, dihydropyrroles **25** were isolated in good yields (Scheme 5) [60]. The incident light raised the photocatalyst to an excited triplet state (PC*) that then transferred an electron to the oxime ethers with production of the radical anions **24** (Scheme 5). Loss of the stable phenolate type anions then occurred with release of the corresponding iminyl radicals that subsequently underwent *5-exo* cyclisation and H-atom transfer with CHD to afford dihydropyrroles **25**.

Furthermore, Leonori and co-workers reported recently that the dye Eosin Y (as PC) catalysed dihydropyrrole formation, simply with light of visible wavelength, when oxime ethers with *O*-2,4-dinitroaryl substitution **27** were employed as reactants [28]. Remarkably, Et_3_N could replace Eosin Y: visible light irradiation of **27** (EWG = 2,4-di-NO_2_) with Et_3_N in CH_3_CN furnished imino-alcohols **31** in yields up to 85%. The complex of Et_3_N with the electron-poor ring of **27**, on excitation with visible light, generated a radical anion that dissociated to give 2,4-dinitrophenoxide together with pyrrolidinylmethyl radical **28**. The oxygen atom was believed to arise from an intermediate such as **29** that fragmented to nitrosophenoxide and a pyrrolidine-containing alkoxyl radical. The latter picked up an H-atom to deliver imino-alcohols **30** as the products (Scheme 5).

**Scheme 6:**
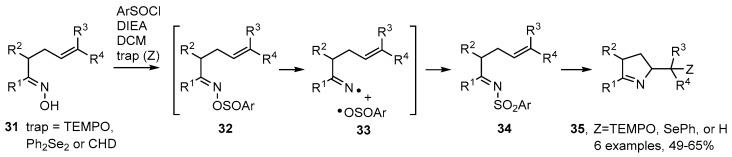
Preparation of dihydropyrroles from *N*-sulfonylimines [61].

Weinreb and co-workers described an alternative strategy in which oximes **31** could be used directly when treated with 2,6-dimethylbenzenesulfinyl chloride in the presence of Hunig’s base (*N,N*-diisopropylethylamine, DIEA) and a radical trapping agent in dichloromethane (DCM) [61]. The best radical traps (Z) were found to be TEMPO and diphenyl diselenide; but CHD could also be used when in great excess (Scheme 6). Probably sulfinate esters **32** were first formed that dissociated to a caged iminyl/sulfonyl radical pair **33**. The reformed *N*-sulfonylimines **34** then released iminyl radicals that cyclised and were trapped to afford functionalised dihydropyrroles **35** in moderate to good yields (Scheme 6).

## 3. Preparations of Pyridine, Quinoline, Phenanthridine and Related Aza-Arenes

Compounds containing the quinoline unit are hugely important to the well-being of society because they play essential roles across the areas of medicine, pharmacology, nutrition, dyes, and even electronics [62]. Bioactive natural products containing quinoline cores are widely distributed in many plants, marine plants, corals, sponges [63,64,65,66,67] and even in chestnut honey [68]. Much the same can be said for compounds containing phenanthridine units. They are noted for their ability to bind to DNA [69] and have antimicrobial, anti-inflammatory, and anti-tumor activities [70,71,72,73,74]. Because of this importance to the field of drug development, synthetic methods for molecules containing these structural units, particularly short, mild and atom-efficient ones, have attracted great attention.

### 3.1. Preparations of Aza-Arenes from Carbonyl Oximes

**Scheme 7:**
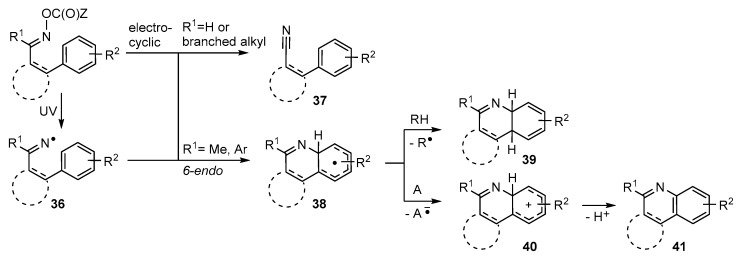
Iminyl radical *6-endo* ring closures onto aromatic acceptors.

Iminyl radicals **36** containing suitably sited aromatic acceptor units usually ring close in *6-endo* mode with formation of a 6-membered N-containing ring **38** (Scheme 7). This rule holds good for cyclisations onto a wide range of aromatic acceptors including benzene, naphthalene, furan, thiophene, indole, pyridine and analogous moieties. As would be expected for less-favoured *6-endo* processes, the rate constants for these iminyl ring closures are significantly smaller than for *5-exo* cyclisations [75]. Never-the-less practicable preparative protocols have been established for many mono- and poly-cyclic heterocycles.

It is usually necessary to work with precursors having the second imine substituent *R*^1^ either Me or Ar. If *R*^1^ is H (or a branched alkyl group) formation of a nitrile **37** is either the main process, or **37** is an important by-product (Scheme 7). Nitrile formation may entail an electrocyclic process of the oxime ester; in which case the proportion may be diminished by suitable solvent choice. With several types of oxime esters cyclisations onto aromatic rings initially create cyclohexadienyl type radicals **38**. If a suitable H-donor is present as solvent, or otherwise, H-atom transfer to radicals **38** yields cyclohexadienes **39** as a mixture of isomers. In practice this route has rarely been developed. Instead restoration of aromaticity to the acceptor ring has usually been observed as in quinoline derivative **41**. This can result when some radical in the system abstracts the highly labile tertiary H-atom of the cyclohexadienyl ring in radical **38**. However, this is a disproportionation that requires two radicals to meet and so is not favored because reactive radical concentrations are always very low. The alternative is that an acceptor molecule A is either added, or is adventitiously present, and single electron transfer (SET) occurs with production of the corresponding cyclohexadienyl cations **40**. These rapidly deprotonate to yield cyclised products **41** with restored aromaticity. This oxidative process is facilitated by non-H-atom donor solvents such as PhCF_3_ or *t*-BuOH.

**Scheme 8:**
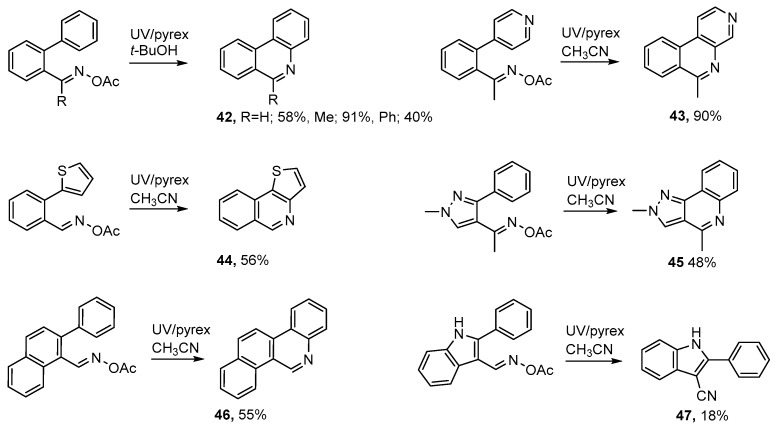
Preparations of tri- and tetra-cyclic heterocycles from acyl oximes [50,52].

Formyl and acyl derivatives of biphenyl, 4-phenylpyridine, 2-phenylthiophene, 3-phenylpyrazole, 2-phenylnaphthalene, 2-phenylindole and analogous aromatics are readily accessible and can be converted to the corresponding acyl oximes without difficulty (see Scheme 8). UV photolyses of these precursors through Pyrex in acetonitrile or *t*-butanol solutions enabled several 6-substituted phenanthridines **42** to be prepared [50,52]. Similarly, benzo[*c*][1,7]naphthyridines **43**, and thieno[3,2-*c*]isoquinolines **44** were conveniently prepared from the corresponding acyloximes.

Analogous photochemical preparations were described for 2,4-dimethyl-*2H*-pyrazolo[4,3-*c*]quinoline **45** and for benzo[*i*]phenanthridine **46**. However, the aldoxime precursor from 2-phenylindole gave only 2-phenyl-*1H*-indole-3-carbonitrile **47** in low yield. Isoquinoline derivatives were also obtained in good yields from photolyses of mixtures of benzophenone acetyl oxime with alkynes in *t*-butanol [50].

Huang, Deng and co-workers recently described a novel method of making 2-arylpyridines by treatment of *O-*acyl oxime esters and acroleins with iodine and triethylamine in toluene at 120 °C [76]. The method had wide scope and yields ranged from moderate to excellent.

**Scheme 9:**
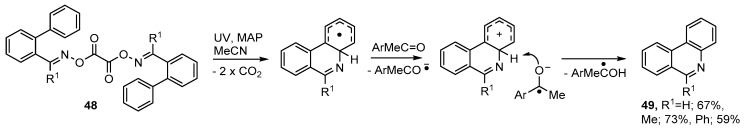
Preparation of phenanthridines from dioxime oxalates [54].

Symmetrical dioxime oxalates containing aromatic acceptors **48** were prepared in essentially quantitative yields, via the oxime oxalyl chlorides, from the corresponding oximes. On UV photolyses at ambient temperature in MeCN solution these returned phenanthridines **49** in clean and atom-efficient processes [54]. Best yields were obtained on inclusion of MAP (ArMeC=O) and it seems this acted as both a photosensitizer and as an electron acceptor to facilitate the final re-aromatization (see Scheme 9).

**Scheme 10:**
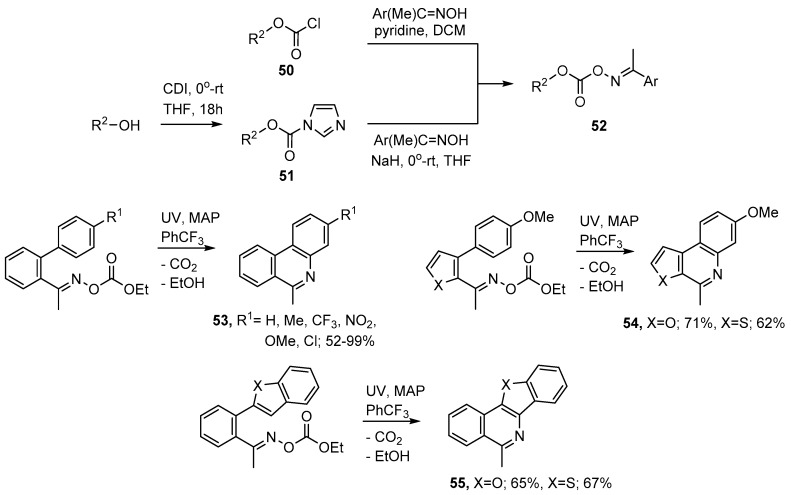
Preparations of aza-arenes from oxime carbonates [75,77].

Oxime carbonates **52** can be prepared either by treatment of an oxime with a chloroformate **50** or via the 1*H*-imidazole-1-carboxylates **51**. The latter can be obtained from reaction of an alcohol with carbonyldiimidazole (CDI) (see Scheme 10). For the preparation of aza-arenes commercial ethyl chloroformate (**50**, *R*^2^ = Et) was found to be very convenient because the main by-product (EtOH) is volatile and easily separated. UV photolyses of appropriately functionalised oxime carbonates, with MAP as additive, enabled phenanthridines **53**, methylfuro- and methylthieno-[2,3-*c*]quinolines **54** to be prepared as well as the 5-methylbenzofuro- and 5-methylbenzo[4,5]thieno-[3,2-*c*]isoquinolines **55** in generally good yields [75,77].

**Scheme 11:**
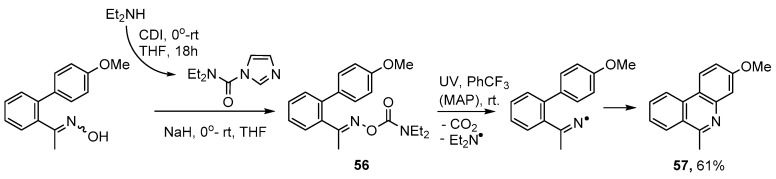
Preparation of phenanthridines from oxime carbamates [78].

Oxime carbamates **56** offer another alternative for aza-arene syntheses but have not yet been much exploited. The Et_2_N aminyl group is sufficiently small that by-products derived from it volatilize away. UV irradiation of **56** in PhCF_3_ at room temperature and with MAP additive provided 3-methoxy-6-methylphenanthridine **57** in 60% yield (Scheme 11) [78]. A slightly higher yield (61%) was obtained in the absence of MAP. This was a worthwhile finding because residues of the photosensitizer can be troublesome to remove.

### 3.2. Preparations of Aza-Arenes from Oxime Ethers

**Scheme 12:**
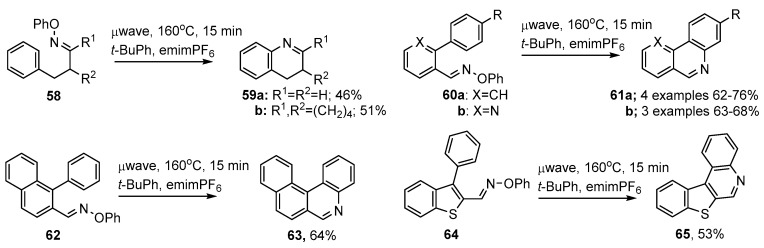
Microwave promoted preparations of aza-arenes from oxime ethers [56,57].

As mentioned above (Section 2.2) *O*-aryl oxime ethers, including those with appropriately placed aryl acceptor units, can easily be made from carbonyl compounds. The 3-phenylpropanone *O*-phenyl oxime ethers **58** were subjected to microwave irradiation at 160 °C in *t*-BuPh together with emimPF_6_ ionic liquid. Quinoline **59a** and tetrahydroacridine **59b** were thereby produced in moderate yields [56,57] (Scheme 12). 

Similarly, biphenyl-2-carbaldehyde *O*-phenyl oximes **60a** and 2-arylnicotinaldehyde *O*-phenyl oximes **60b**, with either electron releasing or electron withdrawing substituents, yielded the corresponding phenanthridine **61a** or benzo[*h*][1,6]naphthyridine derivatives **61b** respectively. The scope of the method was easily extended such that benzo[*k*]phenanthridine **63** and benzo[*b*]thieno[2,3-*c*]quinoline **65** were prepared in a very few steps and in acceptable yields (Scheme 12).

### 3.3. Photoredox Catalyzed Preparations of Aza-Arenes from Oxime Derivatives

**Scheme 13:**
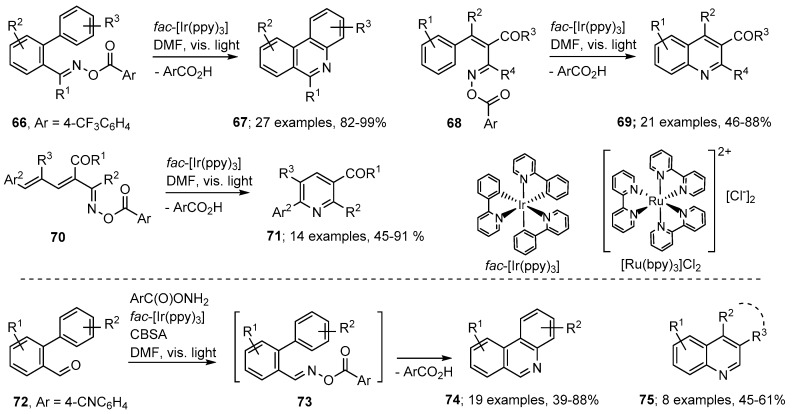
Photoredox methods for preparations of aza-arenes from oxime derivatives [79,80].

The advantages of photoredox catalyzed processes include that visible light, rather than more damaging UV radiation, is usually sufficient and that only catalytic quantities of the PC are required. Several such methods have been reported recently for aza-arene preparations with oxime derivatives as starting materials For example, Zhang, Yu and co-workers generated iminyl radicals from a wide range of aroyl oximes with *fac*-[Ir(ppy)_3_] (ppy = 2-phenylpyridine) as PC [79]. The most reliable and efficient aroyl group was shown to be 4-trifluoromethylbenzoyl; although other electron deficient benzoates succeeded to variable extents. Visible light irradiations of biphenyl aroyl oximes **66** with catalytic quantities of *fac*-[Ir(ppy)_3_] in DMF produced phenanthridine derivatives **67** in very impressive yields (Scheme 13). The PC catalysed the formation of a radical anion from the substrate that then released an iminyl radical together with the 4-trifluorobenzoate anion. The non-H-atom donor solvent DMF favoured production of the fully aromatic products shown. Using similar methodology, the precursors **68** yielded quinolines **69** with a good range of functionality. Furthermore, aroyl oxime esters **70**, with butadienyl substituents, led to iminyl radicals that ring closed selectively in *6-endo* mode with eventual production of pyridine derivatives **71**, generally in very good yields.

Yu and An developed a convenient one-pot tactic that enabled aza-arenes to be prepared in one step from readily available carbonyl compounds [80]. They used *O*-(4-cyanobenzoyl)hydroxylamine [ArC(O)ONH_2_] in conjunction with biphenyl aldehydes **72**. To promote aroyl oxime formation 4-chlorobenzenesulfonic acid (CBSA) was employed as additive. On irradiation with visible light from white LED strips, phenanthridines **74** were obtained directly without isolation of the intermediate oxime esters **73**. A similar one-pot procedure, but employing blue LED strip illumination, was established for preparations of quinoline derivatives **75** from cinnamaldehyde type precursors.

**Scheme 14:**
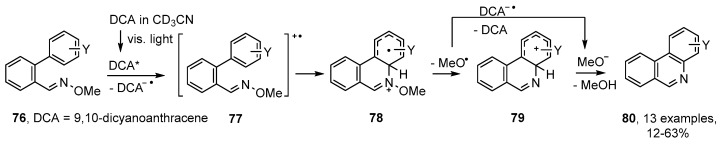
Photoredox production of phenanthridines from 2′-arylbiphenyl oxime ethers [29].

A somewhat related method was reported by de Lijser and co-workers who employed 2′-arylbenzaldehyde oxime ethers **76** together with 9,10-dicyanoanthracene (DCA) [29]. Photolyses at 420 nm in CD_3_CN solutions enabled a set of substituted phenanthridines **80** to be obtained. This process was believed to proceed via radical cation **77** that ring closed by nucleophilic attack of the aromatic unit onto the N-atom of the oxime ether **78** (Scheme 14). Subsequent loss of methoxyl radicals led to cyclohexadienyl cations **79** that underwent deprotonation to afford the product phenanthridines. Yields were variable with strongly electron-releasing substituents Y being deleterious. The main by-product was the volatile and innocuous MeOH and no nitrile formation was observed.

## 4. Preparations of Quinazoline, Quinoxaline and Related Heterocycles from Oxime Derivatives

Bioactivities of various kinds have been associated with numerous substances having structures incorporating quinazoline rings. A number are enzyme inhibitors [81], some display antiviral or anticancer activity [82,83]; others are antibacterial agents [84]. The growing library of approved drugs containing this structural unit, such as erlotinib (Tarceva^®^) [85], prazosin (Minipress^®^, Vasoflex^®^, Lentopres^®^, Hypovase^®^ [86] and gefitinib (Iressa^®^) [87,88], demonstrates the transferable character of aspects of its properties. Steady progress has been made in the development of synthetic methods for quinazoline alkaloids in the last few decades [89,90,91]. Some useful microwave promoted procedures have also been described including those based on: aniline *N*-ethyl carbamates [92], cyano-aromatic compounds with anthranilonitrile [93], *N*-(2-cyanophenyl)-*N,N*-dimethylformamidine derivatives [94], *N*-arylamidines with various aldehydes [95] and 2-aminoaryl imines with aldehydes [96]. The common methods of making the quinazoline ring involve multi-step precursor preparations or special reagents. A pleasing and effective exception is the CuCl_2_-catalysed reaction of aldehydes with anthranilamide [97].

### 4.1. Preparations of Quinazoline Derivatives

**Scheme 15:**
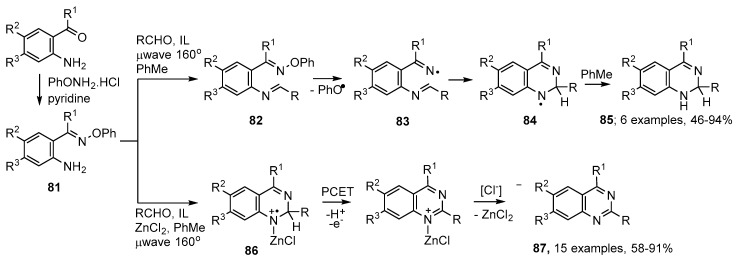
Microwave-assisted preparations of quinazoline derivatives from (2-aminoaryl)alkanone *O*-phenyl oximes [98,99].

Aminoarylalkanone *O*-phenyl oximes **81** can be efficiently prepared from the corresponding ketones (Scheme 15). On mixing these starting materials with an aldehyde and microwave irradiating at 160 °C, in toluene as H-donor solvent, with emimPF_6_ as ionic liquid, imine intermediates **82** were formed. Dissociation to phenoxyl and iminyl radicals **83** took place in one pot, without the need to isolate the imine intermediates **82**. Ring closure was exclusively in the *6-endo* mode with production of aminyl radicals **84** that were reduced to 1,2-dihydroquinazolines **85** by H-atom donation from the solvent [98,99] (Scheme 15).

This proved to be a rapid, mild, reproducible and efficient process with aliphatic, aromatic and heterocyclic aldehydes; the only by-product being phenol. Analogous methodology with **81** and ketones was not successful, except with a few ketones such as cyclohexanone. The dihydroquinazolines **85** slowly oxidized to the corresponding quinazolines in air or oxygen. Alternatively, they could be converted to the latter in high yields by μwave irradiation with DDQ (2,3-dichloro-5,6-dicyanobenzoquinone) at 100 °C in dichloromethane solvent.

Inclusion of ZnCl_2_, a known promoter of imine formation [100], in practice led directly to the corresponding fully aromatic quinazolines **87**. Good to excellent yields were obtained with aliphatic aldehydes, aromatic aldehydes having either electron-withdrawing or electron releasing substituents and with heterocyclic aldehydes. The method was also tolerant of substituents in the aminoaryl rings. It’s likely that, in addition to encouraging imine formation, the ZnCl_2_ forms a complex with the iminyl N-atom in radical cation **86**. The acidity of the H-atom adjacent to the cation would thereby be increased, thus facilitating proton-coupled electron transfer (PCET) and thence to the quinazolines **87** (Scheme 15).

**Scheme 16:**
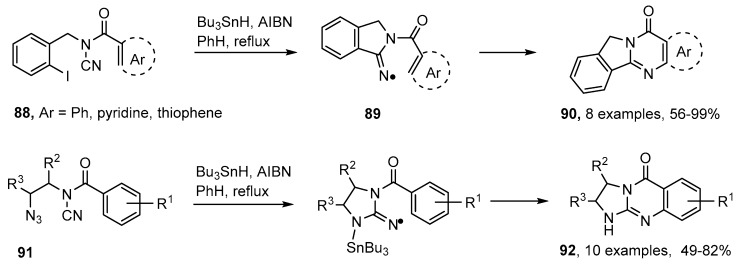
Cascade preparations of quinazolinones and guanidines [105,106,107,108].

Conventional organic preparations of quinazolinones usually start with derivatives of anthranilic acid [89,101]. Several good Pd- and Cu-catalyzed methods for compounds containing this structure have also been published [102,103,104]. Malacria, and co-workers devised an ingenious radical cascade for making quinazolinones starting from *N*-acyl-*N*-(2-iodobenzyl)cyanamides **88** [105,106,107]. Iminyl radicals **89** were obtained by *5-exo*-cyclisation onto the nitrile group of the first-formed aryl radicals. Subsequent ring closure took place efficiently onto a variety of aromatic and heteroaromatic rings yielding quinazolin-2-ones **90** (Scheme 16). The method was subsequently extended to the analogous 2-azidoalkyl-*N*-acylcyanamides **91** and these yielded guanidines **92** [108]. The disadvantages of this methodology were the need to use toxic cyanogen bromide in the syntheses of the precursors and the stoichiometric amounts of organotin hydride needed for the ring closures.

**Scheme 17:**
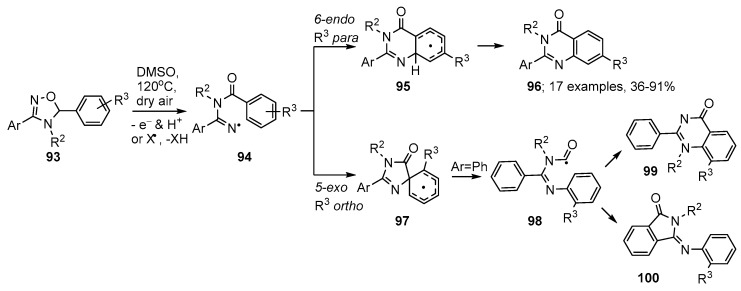
Preparations of quinazolinones from 4,5-dihydro-1,2,4-oxadiazoles [111].

Aware that 5-aryl-4,5-dihydro-1,2,4-oxadiazoles **93** are readily available, either from 1,3-dipolar cycloadditions of nitrile oxides to aldimines, or from reactions of amidoximes with carbonyls [109,110], Chiba and co-workers adopted these as their iminyl radical precursors [111]. They discovered that on treatment in DMSO solution simply with dry air at 120 °C, oxidative rearrangements of **93** were set in motion and resulted in formation of quinazolinones **96** (Scheme 17). Iminyl radicals **94** were generated either via a SET and proton loss process, or by abstraction of the tertiary benzylic H-atom followed by β-scission (Scheme 17). The dominant ring closure was in the *6-endo* mode yielding cyclohexadienyl radicals **95** that were oxidised to the quinazolinone products **96** usually in very good yields. The process was tolerant of electron withdrawing and electron releasing substituents, as well as 5-heteroaryl substituents. Substrates with *ortho-* or *meta*-substituents *R^3^* gave rise to mixtures of isomeric quinazolinones (see structure **99**).

Research with precursor 4,5-dihydro-1,2,4-oxadiazoles with *ortho-R^3^*, or containing 5-pyridine units, indicated that *spiro*-cyclisation to radicals **97** could compete. In this case an alternative ring opening to carbamoyl radicals **98** also took place and these species ring closed either to quinazolinone isomers **99** or in *5-exo*-mode onto a 3-phenyl substituent with production of 3-(arylimino)isoindolin-1-one by-products **100**.

### 4.2. Preparations of Quinoxaline Derivatives

**Scheme 18:**
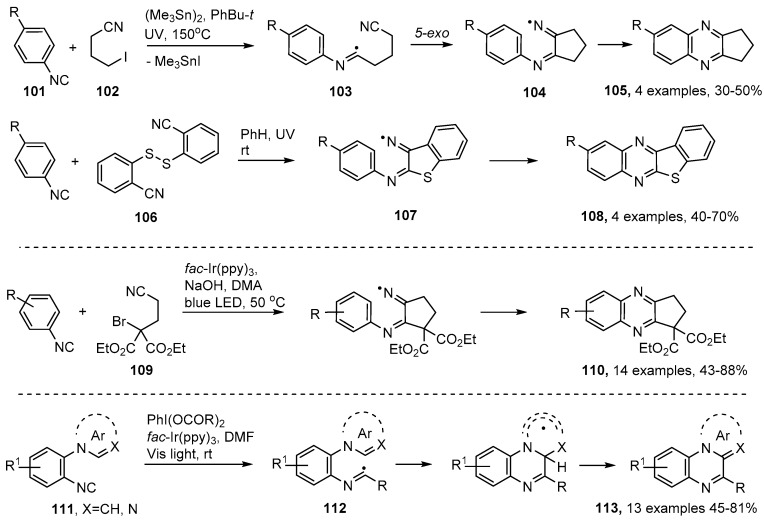
Preparations of quinoxalines via aromatic isonitrile insertion routes [116,117,119].

The quinoxaline and quinoxalin-2-one structural units are also associated with many natural products and biologically active substances [112,113,114,115]. These rings have been assembled in several ways based on initial addition of C-, or S-centered radicals to aromatic isonitriles **101**. Nanni and co-workers generated cyano-substituted alkyl radicals by treatment of 4-iodobutanenitrile **102** with hexamethylditin in *t-*butylbenzene at 150 °C [116]. Additions to the isonitriles generated imidoyl radicals **103** that ring closed onto the pendant cyano group in *5-exo* mode to afford cyclic iminyl radicals **104**. The cascade continued with a further cyclisation on to the aromatic nucleus followed by oxidative re-aromatization to produce cyclopentaquinoxalines **105** in moderate yields (Scheme 18). Significantly, the 2-cyanophenyl disulfide **106** was employed in an analogous process that was free of organotin compounds. UV photolysis of **106**, together with an isonitrile, in benzene solution at r.t. gave rise to benzo[4,5]thieno[2,3-*b*]quinoxalines **108** in fair yields via α-thioimidoyl radicals and polycyclic-iminyl radicals **107** (Scheme 18).

More recently, photoredox catalyzed versions of the isonitrile insertion process requiring only visible light, have been deployed for quinoxaline preparations. Sun *et al.* demonstrated that diethyl 2-bromo-2-(2-isocyanoethyl)malonate **109** was a suitable precursor for 3-cyano-alkyl radicals when irradiated by blue LED light in the presence of *fac*-Ir(ppy)_3_ as PC. Cyclopenta-iminyl radicals were obtained as intermediates and afforded cyclopentaquinoxalines **110** in moderate to excellent yields [117] (Scheme 18). Jamison and co-workers generated alkyl radicals from readily accessible phenyliodine(III) dicarboxylates [118] with visible light and *fac*-Ir(ppy)_3_ in DMF [119]. Their substrates were aromatic isonitriles with *ortho*-pyrrole or analogous heteroarene substituents **111**. The alkyl radicals coupled with the isonitriles giving imidoyl radical intermediates **112** that ring closed to pyrrolo[1,2-*a*]quinoxalines (and related structures) **113** without the intermediacy of iminyl radicals (Scheme 18). The process was tolerant of many substituent types enabling a good range of polycyclic heteroarenes to be prepared. Efficiency was enhanced by their development of a continuous flow system that integrated isonitrile formation with the photo-redox cascade.

## 5. Iminyl Radical Mediated Preparations of Natural Products and Bioactive Compounds

**Scheme 19:**
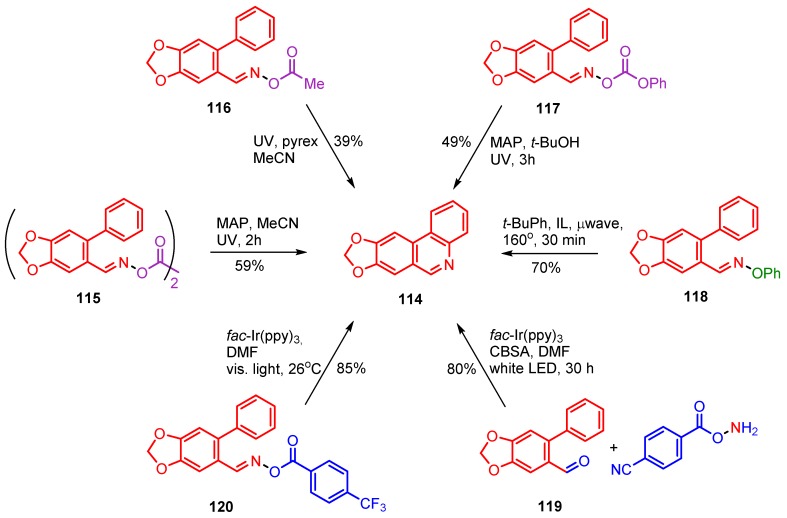
Iminyl radical mediated preparations of trispheridine.

A significant number of iminyl radical based preparative methods have been developed for one or more stages in total or partial syntheses of natural products and bioactive compounds. Trispheridine **114** occurs naturally in flowering plants of the family Amaryllidaceae [120,121]. Trispheridine itself, as well as related compounds, are known to possess anti-tumor activity [122], high anti-retroviral activity [123] and neuro-protective activity [124]. At least six iminyl radical-mediated preparations of trispheridine have been devised and these are summarized in Scheme 19. 

Three of these rely on UVA irradiations; all of easily prepared precursors including dioxime oxalate **115** [54], *O*-acyl oxime ester **116** [52] and *O*-phenyl oxime carbonate **117** [77]. All three precursors were made in high yields, essentially in three steps, from commercially available 6-bromopiperonal. The highest reported yield (59%) was that from dioxime oxalate **115** but it is probable that yields from all three processes could be increased by optimization studies. The μwave promoted process from *O*-phenyl oxime ester **118** was short, clean and comparatively efficient (70%) [57]. Compound **118** was made in high yield in two steps from 6-bromopiperonal and the reaction conditions were clean and straightforward. The two photoredox catalyzed methods starting from aldehyde **119** [80] and trifluoromethylbenzoyl oxime ester **120** [79] had the highest reported yields and reaction conditions were benign (Scheme 19). The *fac*-Ir(ppy)_3_ catalyzed preparation, starting from aldehyde **119**, took only two steps from 6-bromopiperonal. It should be noted, however, that the co-reactant *O*-(4-cyanobenzoyl)hydroxylamine is not commercial; although it can be made in two high-yielding steps from 4-cyanobenzoyl chloride.

**Scheme 20:**
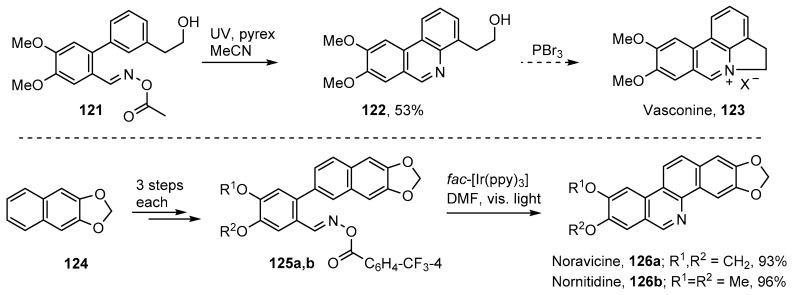
Iminyl radical strategies for vasconine [52], noravicine and nornitidine [79].

Vasconine **123**, along with assoanine, oxoassoanine, pratosine and ismine, constitute another group of alkaloids, isolated from *Narcissus* plants of the Amaryllidaceae family, that contain phenanthridine rings [125,126]. The acyl oxime **121** was targeted as a key compound and prepared in six steps from commercial 2-(3-bromophenyl)ethanol [52]. UV irradiation of this through Pyrex in MeCN solution afforded phenanthridinylethanol derivative **122** in 53% yield (Scheme 20). Compound **122** can be converted to vasconine **123** by treatment with PBr_3_ and can also act as precursor for assoanine, oxoassoanine, and pratosine [125].

Noravicine **126a** and nornitidine **126b** are bioactive benzo[*c*]phenanthridine alkaloids with antitumor activity [127,128]. Zhang, Yu and co-workers devised short (4-steps from methylenedioxy-naphthalene **124**) and high yielding total syntheses employing their photoredox strategy with oxime esters **125a** and **b** [79] (Scheme 20).

Natural products that contain the indolizino[1,2-*b*]quinolin-9(11*H*)-one structure **130a** include camptothecine, mappicine and the nothapodytines [129]. Camptothecine has been obtained from the bark of *Camptotheca acuminata* (*Camptotheca*, Happy tree, Tupelo family) [130] and mappicine from *Mappia foetida miers* (Olacaceae family) [131]. The high bioactivity of these compounds stimulated the production of many synthetic and semi-synthetic analogues [132,133]. Several pharmaceuticals containing this core have been licensed for use including irinotecan (Camptosar^®^, Campto^®^) which is used for the treatment of colon cancer and Topotecan (trade name Hycamtin^®^) which is approved for treatment of several cancers including ovarian and lung types [134]. A number of total syntheses, incorporating important radical-mediated steps, have been reported. Of particular note are those of Curran and co-workers, most of which have key steps involving amidoyl radicals obtained from additions to aromatic isonitriles [135,136,137,138,139,140,141].

**Scheme 21:**
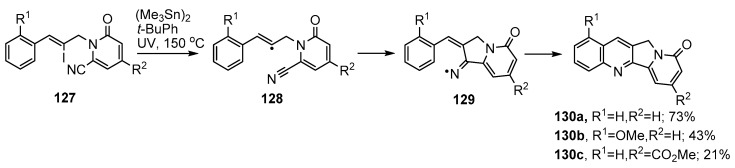
Radical-mediated preparations of indolizino[1,2-*b*]quinolin-9(11*H*)-ones [142,143].

Bowman and co-workers implemented an iminyl radical cascade synthetic protocol for the tetracyclic rings A–D of heterocycles containing the indolizino[1,2-*b*]quinolin-9(11*H*)-one **130a** unit [142,143] (Scheme 21). The starting vinyl iodides **127** were assembled in four steps from cinnamaldehyde derivatives and cyanopyridones and then UV irradiated with hexamethylditin in *t*-BuPh at 150 °C. The initial vinyl radicals **128** ring closed onto the nitrile group in *5-exo* mode producing iminyl radicals **129** that underwent cyclisation and re-aromatization to afford tetracycles **130a**–**c**.

**Scheme 22:**
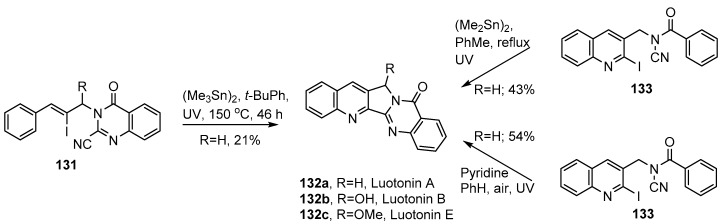
Iminyl radical mediated syntheses of luotonin A [105,107,143].

Luotonins A, B, E (**132a**–**c**) and congeners are structurally related to the mappicines and combine the 2,3-dihydro-1*H*-pyrrolo[3,4-*b*]quinoline ring system with the quinazolinone unit. They have been isolated from plant species of the genus *Peganum* found in China and are well-recognized as remedies in traditional Chinese medicine [144]. They are given for disorders including rheumatism, inflammation, influenza, hepatitis and leukemia and are known antagonists for human DNA topoisomerase I [145,146]. Structure/activity relationships have been investigated for heterocycles of this class [147]. Various non-radical syntheses have been devised [148,149,150,151]. Bowman and co-workers adapted their radical cascade protocol for a comparatively short synthesis of luotonin A **123a** [143]. The vinyl iodide radical precursor **131** was made by alkylation of 4-oxo-3,4-dihydroquinazoline-2-carbonitrile with a substituted cinnamyl bromide. On UV irradiation of a solution of **131** and hexamethylditin in *t*-BuPh, luotonin A **132a** was isolated in a very modest yield of 21% (Scheme 22). A slightly improved yield (30%) was obtained when the reaction was carried out in a sealed tube, at 120 °C, with added di-*t*-butyl peroxide.

Courillon, Malacria and co-workers devised an alternative strategy in which the starting material was the *N*-acyl-*N*-(2-iodobenzyl)cyanamide **133** [105] (Scheme 22). For the luotonin synthesis, 3-(azidomethyl)-2-iodoquinoline was prepared in four steps from quinoline chlorcarbaldehyde and then reduced to the corresponding amine and benzoylated to give **133**. UV irradiation of the latter, with hexamethylditin, in refluxing toluene for 6 h, gave a 43% yield of luotonin A **132a**.

The same research team also discovered an organotin-free protocol employing the same *N*-acyl-*N*-(2-iodobenzyl)cyanamide **133** [107]. When this was UV photolyzed with pyridine (5 equiv.) in refluxing benzene, in air, luotonin A (**132a**) was obtained in an improved yield of 54%.

**Scheme 23:**
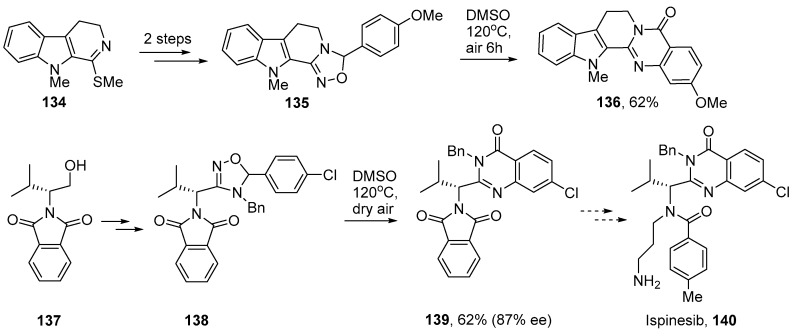
Syntheses of rutaecarpine and ispinesib derivatives via dihydro-1,2,4-oxadiazoles [111].

Chiba and co-workers successfully adapted their dihydro-1,2,4-oxadiazole method for preparations of several bio-active compounds. Rutaecarpine (sometimes rutecarpine) is an indoloquinazoline alkaloid that has been isolated from *Euodia* plants of the Rutaceae family and has non-steroidal anti-inflammatory drug (NSAID) activity [152]. The related 2-methoxy-13-methyl-rutaecarpine **136** has antimalarial activity and has been obtained from *Araliopsis tabouensis*, an African perennial tree [153]. In their synthesis, Chiba and co-workers prepared dihydro-1,2,4-oxadiazole **135** in two steps from indolyl imino methylthioether **134** [111]. Then on heating this as a DMSO solution in air the 2-methoxy-13-methylrutaecarpine **136** was obtained in 62% yield (Scheme 23).

Ispinesib **140** is a synthetic quinazolinone that is under study as a potential anti-cancer agent [154,155]. A similar strategy was employed in a short iminyl radical-mediated synthesis [111]. Alkylated phthalimide **137** was prepared from L-valine and thence to dihydro-1,2,4-oxadiazole **138** as a mixture of diastereoisomers in three steps. On heating one stereoisomer in aerated DMSO the quinazoline derivative **139**, which is a key precursor for ispinesib, was obtained in moderate yield though with some racemization [156]. These dihydro-1,2,4-oxadiazole rearrangements provide a benign, metal-free and atom-economical protocol with great promise as a general synthetic tool for the incorporation of quinazolinone units. 

## 6. Conclusions

Five- and six-membered ring aza-arenes are hugely important in nutrition, medicine and pharmacology because of their many useful therapeutic properties. Short, mild and atom-efficient synthetic methods for compounds containing these units are clearly provided by iminyl radical mediated systems. Although iminyl radicals can be accessed by a variety of ways, carbonyl oximes and oxime ethers are particularly effective non-toxic precursors with long shelf lives. These starting materials lend themselves to ‘clean’ preparative protocols, free of harsh acid/base or redox reagents, and employing photolysis, μwave irradiation or photoredox catalysis. Iminyl radicals generated by these means and suitably substituted with acceptor units facilitate syntheses of a large variety of such compounds. Suitable acceptor groups include aromatics, heteroarenes, alkenes and alkynes. Reproducible and efficient syntheses of good scope and impressive generality have thereby been devised for diverse families of heterocycles. Significantly, iminyl radical based steps have been chosen for natural products and bioactive molecules containing phenanthridine, indole and quinazoline core units. It seems certain that further variations of these methods will be devised and find additional roles and applications. It can be reliably concluded that iminyl-radical based synthetic methods form a most valuable toolkit to expedite the exploration of ‘drug-like’ chemical space.
